# A Multi-OMICs Approach Sheds Light on the Higher Yield Phenotype and Enhanced Abiotic Stress Tolerance in Tobacco Lines Expressing the Carrot *lycopene* β*-cyclase1* Gene

**DOI:** 10.3389/fpls.2021.624365

**Published:** 2021-02-05

**Authors:** Juan C. Moreno, Silvia Martinez-Jaime, Monika Kosmacz, Ewelina M. Sokolowska, Philipp Schulz, Axel Fischer, Urszula Luzarowska, Michel Havaux, Aleksandra Skirycz

**Affiliations:** ^1^Max Planck Institut für Molekulare Pflanzenphysiologie, Potsdam, Germany; ^2^Biological and Environmental Science and Engineering Division, Center for Desert Agriculture, King Abdullah University of Science and Technology, Thuwal, Saudi Arabia; ^3^Aix-Marseille Univ., CEA, CNRS UMR7265, BIAM, CEA/Cadarache, Saint-Paul-lez-Durance, France; ^4^Boyce Thompson Institute, Cornell University, Ithaca, NY, United States

**Keywords:** β-carotene, carotenoids, abiotic stress, lycopene β-cyclase, omics, ROS, *Nicotiana tabacum* cv Xanthi, transcription factors

## Abstract

Recently, we published a set of tobacco lines expressing the *Daucus carota* (carrot) *DcLCYB1* gene with accelerated development, increased carotenoid content, photosynthetic efficiency, and yield. Because of this development, *DcLCYB1* expression might be of general interest in crop species as a strategy to accelerate development and increase biomass production under field conditions. However, to follow this path, a better understanding of the molecular basis of this phenotype is essential. Here, we combine OMICs (RNAseq, proteomics, and metabolomics) approaches to advance our understanding of the broader effect of *LCYB* expression on the tobacco transcriptome and metabolism. Upon *DcLCYB1* expression, the tobacco transcriptome (~2,000 genes), proteome (~700 proteins), and metabolome (26 metabolites) showed a high number of changes in the genes involved in metabolic processes related to cell wall, lipids, glycolysis, and secondary metabolism. Gene and protein networks revealed clusters of interacting genes and proteins mainly involved in ribosome and RNA metabolism and translation. In addition, abiotic stress-related genes and proteins were mainly upregulated in the transgenic lines. This was well in line with an enhanced stress (high light, salt, and H_2_O_2_) tolerance response in all the transgenic lines compared with the wild type. Altogether, our results show an extended and coordinated response beyond the chloroplast (nucleus and cytosol) at the transcriptome, proteome, and metabolome levels, supporting enhanced plant growth under normal and stress conditions. This final evidence completes the set of benefits conferred by the expression of the *DcLCYB1* gene, making it a very promising bioengineering tool to generate super crops.

## Introduction

Carotenoids are C40 isoprenoid compounds synthesized in the plastids of photosynthetic and some nonphotosynthetic organisms (e.g., plants, algae, fungi, and bacteria) (Ruiz-Sola and Rodriguez-Concepcion, 2012). Carotenoid biosynthesis pathway is well-known and has been characterized in many plant species (Fraser et al., 1994; Moise et al., 2014; Nisar et al., 2015). Carotenoid synthesis is of great importance for plant physiology because of its important functions in photosynthesis and photoprotection (Niyogi et al., 1997; Holt et al., 2005), pollination (Bartley and Scolnik, 1995), scavenging of reactive oxygen species (ROS), and indirectly in hormone biosynthesis (serving as the precursors of abscisic acid/ABA and strigolactones/SLs) (Schwartz et al., 1997; Alder et al., 2012). In mammals, carotenoids (especially β-carotene) serve as a dietary precursor of vitamin A, which is required for the maintenance of normal vision, healthy immunity, and cell growth (Olson, 1996). In addition, carotenoids (e.g., β-carotene) have been shown to have antioxidant-promoting activities in humans (Fraser and Bramley, 2004; Rao and Rao, 2007). These properties make β-carotene a very valuable molecule for plant functioning, but also for improving food quality content in crops. Because of this, genetic engineering approaches have been used to increase the β-carotene content in several plant models by expressing the lycopene β-cyclase (*LCYB*) gene, which encodes for the enzyme (LCYB) involved in its production. For instance, (over)expression of the *LCYB* gene in Arabidopsis, tomato, and sweet potato leads to increased tolerance to abiotic stresses, such as salt and drought (D'Ambrosio et al., 2004; Chen et al., 2011; Kang et al., 2018). Moreover, increases in β-carotene, violaxanthin, zeaxanthin, and lutein have been reported to increase plant tolerance to high light, UV irradiation, and salt through scavenging ROS (Davison et al., 2002; Gotz et al., 2002; Han et al., 2008; Shi et al., 2015; Kang et al., 2018). Interestingly, in tobacco, carrot, and sweet potato *LCYB*-expressing lines, a carotenoid increase was accompanied by an induction in carotenoid genes such as phytoene synthase (*PSY*), phytoene desaturase (*PDS*), ζ-carotene desaturase (*ZDS*), zeaxanthin epoxidase (*ZEP*), violaxanthin de-epoxidase (*VDE*), and neoxanthin synthase (*NXS*) (Moreno et al., 2013; Shi et al., 2015; Kang et al., 2018); thus, this suggests the possibility that an additional signal coordinates the expression of carotenoid genes. Previous studies have shown that the coordinated expression of carotenogenic genes tightly regulates carotenoid metabolism (Liu et al., 2015; Yuan et al., 2015). However, knowledge of the transcriptional regulatory mechanisms controlling the expression of these genes is limited. Several studies have shown numerous transcription factors influencing carotenoid accumulation in plants. For instance, the MADS-box genes *AGAMOUS-like 1* and *FRUITFULL* regulate carotenoid accumulation during tomato fruit ripening (Vrebalov et al., 2009). Moreover, tomato ripening inhibitor (RIN) regulates carotenoid accumulation via binding to the promoter region of the *PSY* gene (Martel et al., 2011). Other transcription factors belonging to the AP2/ERF (Welsch et al., 2007; Chung et al., 2010; Lee et al., 2012), NAC (Ma et al., 2014; Zhu et al., 2014a,b), and MYB subgroups (Sagawa et al., 2016; Zhu et al., 2017) modulate fruit ripening and carotenoid accumulation in several plant models. Intriguingly, the WD40 and bZIP TF families have been reported to be involved in the control of multiple biological processes, including plant growth and development, fruit ripening, and stress responses (Smith et al., 1999; Jain and Pandey, 2018). Furthermore, a kiwi R2R3 MYB (*AdMYB7*) that binds to and activates the expression of *LCYB* (and chlorophyll biosynthetic genes) is responsible for chlorophyll and carotenoid accumulation in kiwi (Ampomah-Dwamena et al., 2019).

Recently, the expression of the *LCYB* gene from carrots (*DcLCYB1*) in tobacco resulted in an increase in pigment content (β-carotene and chlorophylls), gibberellin (GA_4_), and plant biomass in T1 tobacco lines grown under controlled conditions (Moreno et al., 2016). This positive effect was confirmed to be stable through generations (T4–T5) and in plants grown under fully controlled (constant and fluctuating light regimes) and noncontrolled climate conditions. Moreover, an increased gibberellin (GA)/ABA ratio (along with increased carotenogenic gene expression and pigment accumulation) was associated with higher fitness, yield, and photosynthetic efficiency in these transgenic tobacco lines (Moreno et al., 2020). Intriguingly, we did not observe any trade-off or detrimental effect upon the *DcLCYB1* expression in our tobacco plants. However, we observed a general induction in the expression of key genes from several plastid pathways, such as GAs, chlorophyll, and carotenoids.

Over the past two decades, with the sequencing of entire genomes and the establishment of high-throughput methodologies for gene expression analysis, plant research has entered the genomic era (Leister, 2003). In recent years, with the peak of functional genomics (analysis of transcriptome, proteome, and metabolome), plant research has started to explore new gene, protein, and metabolite functions by the characterization of new pathways and new components of previously known pathways (Moreno, 2019). Here, we apply a multi-OMICs (RNAseq, proteomics, and metabolomics) approach and stress treatments to fully characterize the general plant response (beyond carotenogenesis and isoprenoid pathways) at molecular and physiological level in the tobacco *DcLCYB1* lines.

## Materials and Methods

### Plant Material and Growth Conditions

Tobacco transgenic lines corresponding to the T4 generation of our previously published *DcLCYB1*-expressing lines were used in the current work (Moreno et al., 2016). Tobacco (*Nicotiana tabacum* cultivar Xanthi NN) wild type and transgenic lines were raised from seeds germinated in Petri dishes containing an MS medium supplemented with 30 g/L sucrose (Murashige and Skoog, 1962) and kanamycin (100 mg/mL). Radicle emergence and germination experiments were performed in Petri dishes supplemented with 3% sucrose (*n* = 20, experiment was repeated three times in three different Petri dishes). Leaf generations and internode length of 40-day-old tobacco wild type and transgenic lines were measured. ImageJ software was used to quantify the leaf area (*n* = 3).

### RNA Isolation

Total RNA was extracted from the frozen powder of tobacco leaves of 5-week-old T_3_ plants (three biological replicates per lines, *n* = 3) using the Nucleo Spin RNA extraction kit (Macherey-Nagel, Germany). Genomic DNA traces were eliminated by a 15 min DNase I treatment.

### RNA Sequencing (RNAseq) and Data Analysis

QuantSeq 3' mRNA-Seq services were performed for the wild type and transgenic *DcLCYB1* lines L14, L15, and L16 (*n* = 3 biological replicates per genotype) at Lexogen GmbH (Vienna, Austria). Here, 500 ng total RNA were used as input for generating sequencing-ready libraries using the QuantSeq FWD 3' mRNA-Seq library preparation kit (Lexogen, Vienna, Austria; SKU 015). The libraries were characterized by microcapillary electrophoresis and fluorometry prior to pooling and sequencing using the same methods on an Illumina NextSeq500. A sequencing yield of 572.9 M demultiplexed reads was obtained from a single run in SR75 HO mode. An integrated data analysis was performed by Lexogen (STAR aligner), where the reads were mapped against the NiTAB 4.5 reference genome, read-counts were determined, and differential expression was computed using DESeq2 in R (Love et al., 2014). In addition, NiTAB 4.5 identifiers were mapped in house (Max Planck Institute of Molecular Plant Physiology) to *Arabidopsis* genome, obtaining ~25,000 mapped genes (out of 36951 NiTAB 4.5 identifiers). Lexogen GmbH (Vienna, Austria) provided the principal component analysis (PCA) analysis of the data and statistical tests. Localization analyses were performed using the SUBA3 database (Tanz et al., 2013). Venn diagrams were designed, including for all the genes that changed significantly (padjust < 0.05) in the transgenic lines. The N.A. values were removed from the data set, and only NiTABs properly mapped to AGIs were used. Because of the tetraploid origin of tobacco, several NiTABs could be mapped to the same AGI; therefore, for the Venn diagrams, those NiTABs were treated as one [e.g., L14 has only 2,138 unique AGIs (out of 2,624), L15 (2,428 out of 2,998), and L16 (3,792 out of 4,958)]. A fold enrichment analysis (Fisher's exact test with FDR correction *p* < 0.05) was performed using the PANTHER overrepresentation test (PANTHER13.1) with the GO ontology database released on 2018.02.02, here with the annotation data set GO biological process complete (Mi et al., 2017). The reference list corresponds to all annotated *Arabidopsis* genes, and our data set corresponds to the up- or downregulated genes in our analysis. A Fisher's exact test with FDR multiple test correction (*p* < 0.05) was used to select the up- and downregulated genes. Raw sequencing data were deposited at gene expression omnibus (GEO) from NCBI under project accession number GSE157541.

### MapMan Analysis of the RNAseq Data

**Figure 1:**
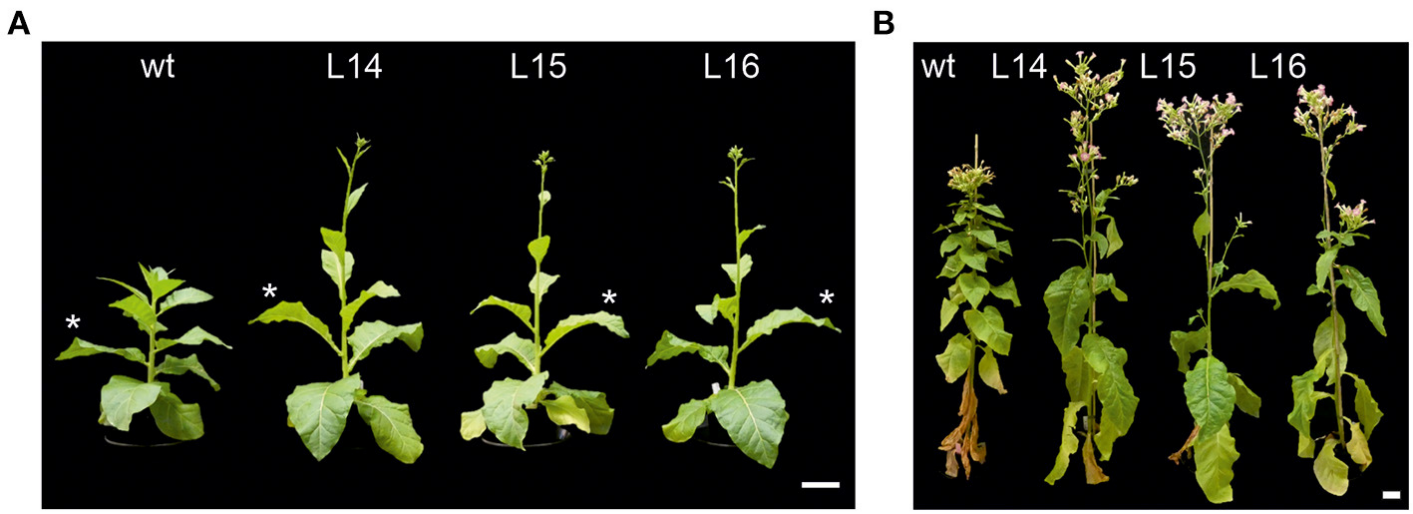
Plant phenotype of transgenic *DcLCYB1*-expressing lines. **(A,B)** Six- (left panel) and 16-week-old (right panel) wild type and transgenic *DcLCYB1* plants (L14, L15, and L16) grown under semicontrolled conditions in the greenhouse (light intensity: 144–1,000 μE m^−2^ s^−1^; temperature: 20–28°C; R.H. 64%). Asterisks show the fourth leaf selected for each plant for further OMICs experiments. Scale bars: 10 cm. wt: wild type.

As input data for MapMan (Thimm et al., 2004), we selected genes changing significantly in at least two of the three transgenic lines (all overlaps between L14 and at least other transgenic line), and we chose the L14 (1,920 genes) expression data to visualize in the software. In this way, the majority of affected processes (comprising 1,542 genes) in all three lines are shown in the figure plus additional processes (comprising 382 genes) affected in at least 2 transgenic lines. We have selected line L14 because of its higher plant height (Figure 1) and biomass production (Moreno et al., 2020) but also to simplify the visualization of the data.

### Protein Extraction, Trypsin Digestion, and Mass Spectrometric Analyses

Protein extraction (whole-leaf proteome) and further protein digestion were performed as described in Moreno et al. (2018) but only using 50 μg of the total protein for digestion. In brief, after total protein extraction, each sample (*n* = 3 biological replicates) was subjected to the filter-aid sample preparation (FASP) procedure (Wisniewski et al., 2009). Peptide purification was performed using SepPack columns (SPEC18 100 mg mL21; Teknokroma), and samples were dried in a SpeedVac for 5 h and kept frozen until use for mass spectrometry.

### Liquid Chromatography-Mass Spectrometry

Prior to analysis peptides were resuspended in 50 μl of resuspension buffer [3% (v/v) acetonitrile, 2% (v/v) TFA]. Peptides were measured by the Q-Exactive HF (Thermo Scientific) high-resolution mass spectrometer coupled to ACQUITY UPLC M-Class system (Waters). Samples were separated by reverse-phase nano liquid chromatography in 120 min, gradient ramped from 3.2% ACN to 7.2% ACN over first 20 min then gradient increase to 24.8% ACN over next 70 min and to 35.2% ACN over 30 min, followed by a 5 min washout with 76% ACN. The MS was run using a data dependent top-N method that fragmented the top 15 most intense ions per full scan. Full scans were acquired at a resolution of 120,000 with an AGC target 3e6, maximum injection time 50 ms, scan range 300–1,600 m/z. Each dd-MS2 scan was recorded in profile mode at a resolution of 30,000 with an AGC target of 1e5, maximum injection time 100 ms, isolation window 1.2 m/z, normalized collision energy 27 and the dynamic exclusion set for 30 s.

### Protein Identification and Label Free Quantitation

DDA raw MS/MS spectra was processed with MaxQuant (version 1.6) for protein identification and quantitation (Cox and Mann, 2008). Peptide identification by the Andromeda search engine was based on the in-house POTbaseMS database (Moreno et al., 2018). The following parameters were applied to the analysis: 10 ppm peptide mass tolerance; 0.8 Da MS/MS tolerance; Trypsin was specified as enzyme and a maximum of two missed cleavages were allowed; a decoy database search with a 1% FDR cutoff on the peptide and protein level; carbamidomethylation of cysteine was set as a fixed modification, while the oxidation of methionine was set as variable modification. The “label-free quantification” and “match between runs” settings were also highlighted in the software. Valid peptides were expected to have a minimum length of six amino acids. Peptide quantitation was performed for proteins identified with at least two peptides (a minimum of one unique and one razor) unmodified peptide. Peptides intensity was taken and further normalized by LFQ algorithm. Known contaminants and reversed hits were removed from the analysis.

### Statistical Analysis and Data Processing of the Proteomics Data

Protein intensity was log2 transformed and the linear model for microarray analysis (limma) R package (Ritchie et al., 2015) was used for comparative analysis (*p* ≤ 0.05). Limma offers robust differential analysis of data with missing values, common in DDA methods of label-free MS experiments (Lazar et al., 2016). PCA was implemented by using prcomp function and was visualized using ggbiplot R package. Arabidopsis ATG codes were mapped to the identified POTs, where each POT can comprise one or more Arabidopsis ATGs. A fold enrichment analysis (Fisher's exact test with FDR correction *p* < 0.05) was performed using PANTHER, as explained above. Arabidopsis ATG codes mapped to each POT were used for this analysis. The mass spectrometry proteomics data have been deposited to the ProteomeXchange Consortium via the PRIDE (Perez-Riverol et al., 2019) partner repository with the dataset identifier PXD023595.

### Gene and Protein Network Analysis

Gene and protein network analyses were performed using the String database (Szklarczyk et al., 2017), and Cytoscape was used for a network visualization (Shannon et al., 2003). For gene networks, we selected the genes changing significantly in at least two of the three transgenic lines and used the String database for the gene–gene interaction analysis. The network edges represent the confidence of the data extracted from published experiments and databases. The highest interaction score was set at 0.900, which is the highest confidence parameter for the analysis. Disconnected nodes in the network were omitted. The interaction values were extracted and exported as an Excel table and then used as an input table for network visualization in Cytoscape. The obtained Cytoscape network was divided into clusters by using the ClusterOne app (set up with the standard parameters) in Cytoscape (51 clusters comprising 419 interacting genes). The same pipeline and parameters were used to build the protein–protein interaction network (410 proteins changing significantly in at least two lines) and to perform the cluster analysis (19 clusters). The cluster names were assigned by using the enrichment analysis provided by the String software (e.g., GO function, GO biological process, KEGG pathway).

### Extraction and Phase Separation for LC-MS Analyses

A methyl *tert*-butyl ether (MTBE) extraction buffer was prepared, and the samples (*n* = 6 biological replicates) were subjected to the extraction method described in Salem et al. (2016).

### Secondary Metabolite Measurements

The dried aqueous phase (300 μL), which contained the secondary metabolites, was measured using ultra-performance liquid chromatography coupled to an Exactive mass spectrometer (Thermo-Fisher Scientific) in positive and negative ionization modes, as described in Giavalisco et al. (2011). The LC-MS data were processed using Expressionist Refiner MS 11.0 (Genedata AG, Basel, Switzerland). The settings were as follows: chromatogram alignment (RT search interval 0.5 min), peak detection (summation window 0.09 min, minimum peak size 0.03 min, gap/peak ratio 50%, smoothing window five points, center computation by intensity-weighted method with threshold at 70%, boundary determination using inflection points), isotope clustering (RT tolerance at 0.015 min, m/z tolerance 5 ppm, allowed charges 1–5), filtering for a single peak not assigned to an isotope cluster, adduct detection, and clusters grouping (RT tolerance 0.05 min, m/z tolerance 5 ppm, maximum intensity of side adduct 100,000%). All metabolite clusters were matched to the in-house libraries of authentic reference compounds, allowing a 10 ppm mass and dynamic retention time deviation (maximum 0.2 min).

### Targeted Lipid Profiling by LC-MS and Data Analysis

The measurement of the lipids from *N. tabacum* leaves was performed as described by Hummel et al. (2011). In brief, the dried organic phase was measured using a Waters Acquity ultra-performance liquid chromatography system (Waters, http://www.waters.com) coupled with Fourier transform mass spectrometry (UPLC-FT-MS) in positive and negative ionization modes. The analysis and processing of the mass spectrometry data was performed with REFINER MS® 10.0 (Gene Data, http://www.genedata.com) and comprised peak detection, RT alignment, and chemical noise removal. The derived mass features, characterized by specific peak ID, m/z values, retention time, and intensity, were further processed using custom R scripts. Prior to annotation of the metabolic features using the in-house lipid database, isotopic peaks were removed from the MS data. Annotated lipids were confirmed by manual investigation of the chromatograms using Xcalibur (Version 3.0, Thermo-Fisher, Bremen, Germany). The database used in this project includes 279 lipid species. The peak intensities were day-normalized, sample median-normalized, and, subsequently, log-2 transformed. The resulting data matrices were used for further peak filtering and analysis in Excel (Microsoft, http://www.microsoft.com). Significant differences were determined using a nonpaired two-tailed student *t*-test (*p* < 0.05).

### Photooxidative Stress

Leaf discz of a 1.2 cm diameter floating on water at 10°C were exposed for 18 h to a strong white light (PFD, 1,200 mmol photons m^−2^ s^−1^) that was produced by an array of light-emitting diodes. Autoluminescence emission from the stressed leaf discz placed on wet filter paper was measured after 2 h of dark adaptation, as previously described (Birtic et al., 2011). The signal was imaged with a liquid nitrogen–cooled CCD camera (VersArray 1300B, Roper Scientific), with the sensor operating at a temperature of −110°C. The acquisition time was 20 min, and on-CCD binning of 2 × 2 was used, leading to a resolution of 650 × 670 pixels. As previously shown, the imaged signal principally emanates from the slow decomposition of lipid peroxides, which accumulated in the samples during the oxidative stress treatment (Birtic et al., 2011).

### Oxidative and Salt Stress Experiments

Transgenic and wild type tobacco seeds (T4 generation) were sterilized and germinated on solid MS medium supplemented with 3% sucrose. After 12 days, seedlings were transferred to a 24-well plate, including six biological replicates of each genotype (*n* = 6). Seedlings were grown for seven days in liquid MS medium containing either mock (H_2_O), H_2_O_2_ (50 mM) or catechin (0.175 mM), and kept on a horizontal orbital shaker (120 rpm), under constant light (80 μE m^−2^ s^−1^, 22°C). Fresh weight was recorded after 7 days for each plant. For salt stress experiments tobacco seeds (T4 generation) were germinated on solid MS media (3% sucrose) supplemented with 0, 100, and 150 mM of NaCl. Fresh weight was recorded after 3 weeks (*n* = 8–9; experiment was repeated twice).

## Results

### RNAseq Revealed the Broader Impact of *DcLCYB1* Expression on the Transcriptome of Tobacco Plants With Increased Plant Yield

Our tobacco *DcLCYB1*-expressing lines showed a growth phenotype characterized by bigger leaves, long internode spaces, and early development (Moreno et al., 2020) (Figure 1A). Even at later stages of the tobacco life cycle (16-week-old plants), the transgenic lines L14, L15, and L16 showed higher plant height than the wild type (Figure 1B). Moreover, among the transgenic lines, L14 was the one that showed the best performance (Figure 1B). In our previous transcript analysis (qRT-PCR), most of the analyzed genes (e.g., carotenoid or carotenoid-related pathways and photosynthesis-related genes) were upregulated (Moreno et al., 2020). These genes were selected based on previous evidence of their carotenoid and/or carotenoid-related function (Moreno et al., 2020). However, we believe there has to be a reshaping and a “trade-off” of gene expression in the tobacco genome that supports a greater plant yield.

**Figure 2:**
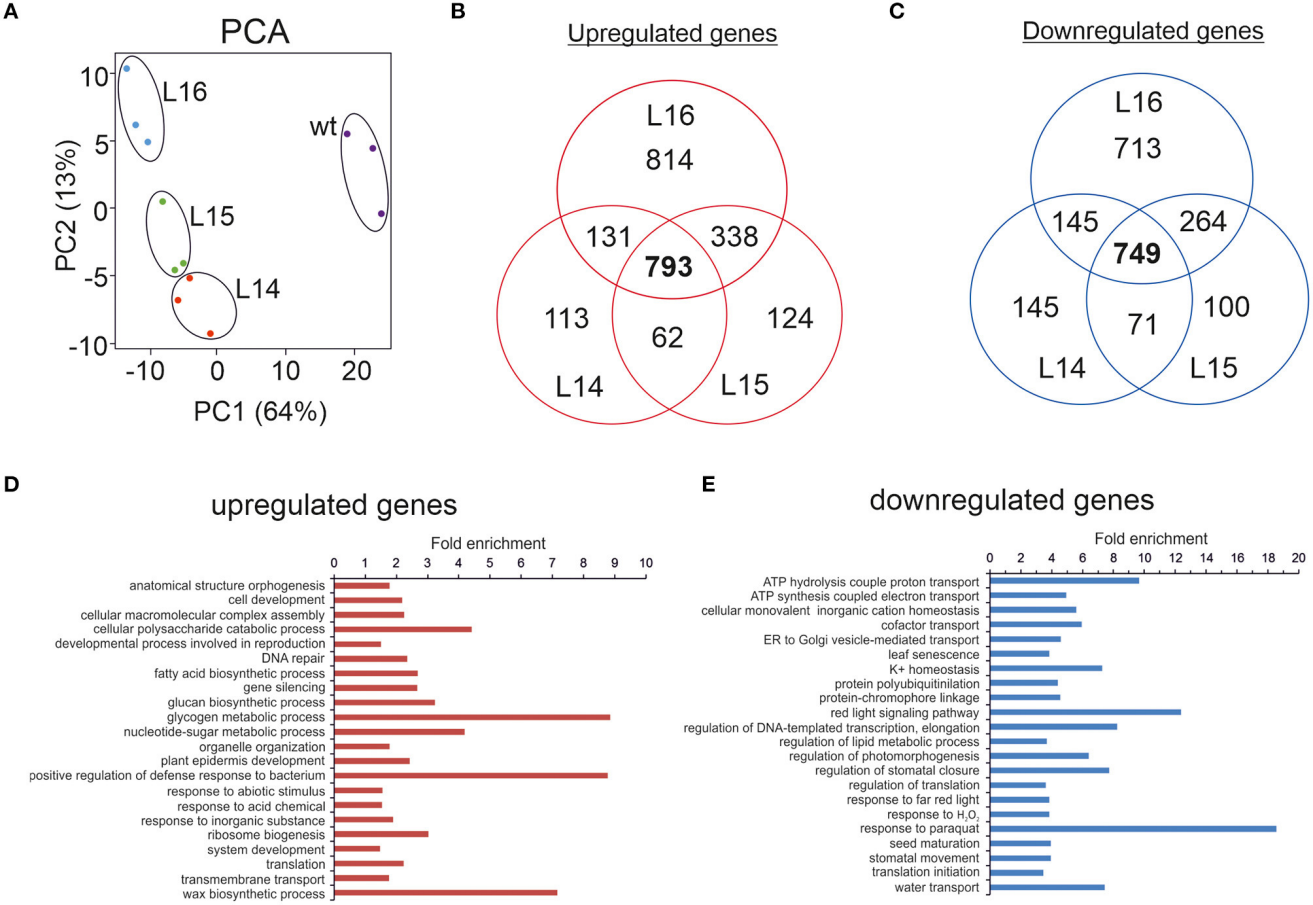
RNA sequencing of transgenic *DcLCYB1* lines. **(A)** Principal component analysis (PCA). **(B,C)** Venn diagram of up- and downregulated genes in the L14, L15, and L16 transgenic lines. **(D,E)** Fold enrichment analysis of processes (GO terms) containing the up- and downregulated genes present in the *DcLCYB1* line L14. Three biological replicates for each genotype (wt, L14, L15, and L16) were used for RNAseq (*n* = 3).

To obtain further insights into the additional genes or pathways that support growth in these tobacco lines, we performed RNAseq analysis and compared the transcriptome of the transgenic lines with the wild type (Figure 2). In our RNAseq experiment, the PCA showed how similar the replicates are between genotypes and also showed the differences between the wild type and the transgenic lines (Figure 2A). In addition, L14 and L15 were more similar than line L16. To better analyze and visualize our RNAseq results, the NiTAB identifiers (~37,000) were annotated into *Arabidopsis* identifiers (e.g., At2g30390; see the Material and Methods section). Most of the proteins encoded by all the identified genes (~25,000) that we could reliably annotate in *Arabidopsis* were localized across all cell compartments (SUBA3 database; Supplementary Figure 1; Supplementary Table 1). From those compartments, the nucleus, cytosol, and mitochondria showed the highest location frequency of genes in our data [excluding the not annotated (N.A.) group]. Interestingly, the highest localization frequency of differentially expressed genes (DEGs; upon *DcLCYB1* expression) was observed in the nucleus, cytosol, and chloroplast (Supplementary Figure 1) for lines L14 (2,624 genes), L15 (2,998 genes), and L16 (4,958 genes). Upregulated genes for lines L14 (1,278), L15 (1,557), and L16 (2,609) showed similar location frequency (in percentage) across the 11 cell compartments (Supplementary Figure 1). The same pattern was observed for downregulated genes in lines L14 (1,346), L15 (1,441), and L16 (2,349) across the same compartments (Supplementary Figure 1).

To narrow down our analysis, we built Venn diagrams with the DEGs in the transgenic lines (Figures 2B,C). In this case, we used only NiTAB identifiers with their mapped AGI code; the N.A. values were removed from our data set. In addition, because of the tetraploid origin of tobacco, several NiTAB identifiers could have been mapped to the same AGI code in Arabidopsis; therefore, those were treated as one for the Venn diagram analysis. Thus, the number of DEGs was reduced for L14 (2,138), L15 (2,428), and L16 (3,792). The Venn diagrams show an overlap of 793 and 749 upregulated and downregulated genes, respectively, for the three transgenic lines (Figures 2B,C). An enrichment analysis of processes (Fisher's exact test with FDR correction *p* < 0.05) were performed using the PANTHER overrepresentation test (PANTHER13.1) with the GO ontology database (Mi et al., 2017). The upregulated genes classified in processes such as glycogen metabolic process, positive regulation of defense responses to bacterium, and wax biosynthetic process showed 7–9-fold enrichment, while the downregulated genes belonging to processes such as response to paraquat, red light signaling pathway, and ATP hydrolysis coupled to proton transport showed 9–18-fold enrichment (Figures 2D,E). Other processes with at least 2-fold enrichment are also shown (Figures 2D,E).

### MapMan and Gene Network Analysis Reveal Enhanced Stress Response and Processes Related to Translation, Ribosome, and RNA Metabolism

**Figure 3:**
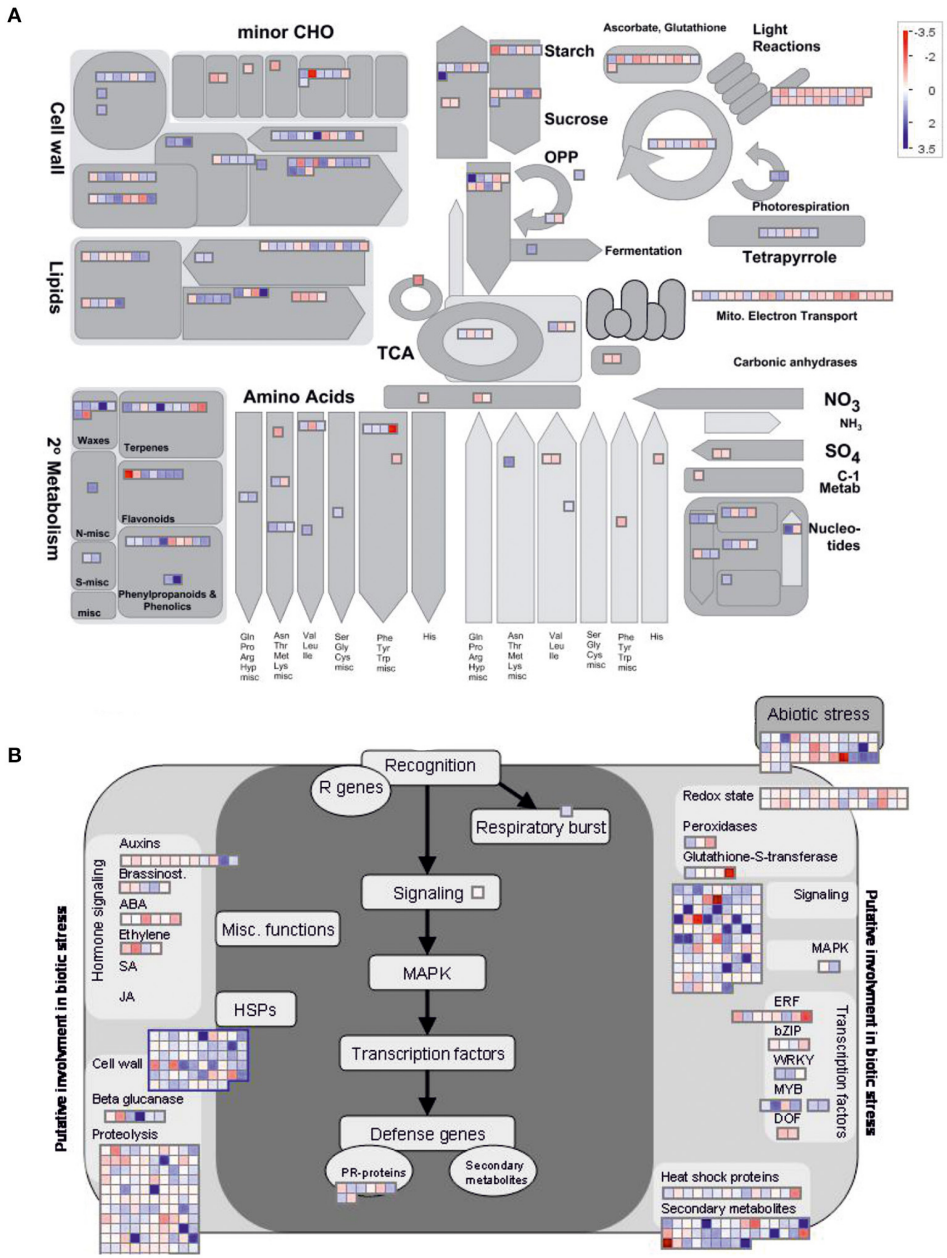
MapMan representation of transcriptional perturbations in *DcLCYB1*-expressing plants. **(A)** Metabolism overview of the schematic representation generated in MapMan depicting metabolic perturbations. More than 300 genes (317) involved in different metabolic processes were significantly changed in the analysis. **(B)** Biotic stress schematic representation generated in MapMan depicting the changes in the genes involved in biotic and abiotic stress. More than 450 genes (469) involved in different metabolic processes were significantly changed in the analysis. Log2 fold change expression data were used.

To gain further insights into the pathways that might be affected by *DcLCYB1* expression in the transgenic lines, we used MapMan software for better visualization of our data set (see the Material and Methods section). To simplify the analysis, we decided to use the significant changes occurring in line L14 because of its higher fitness (Figure 1) and biomass production (Moreno et al., 2020). Because of the high number of DEGs analyzed in Thimm et al. (2004), they were classified across 34 out of 36 MapMan Bins (Supplementary Table 2). The top five bins are number 29 (Protein metabolism) with 314 genes, 27 (RNA metabolism) with 204 genes, 34 (transport) with 111 genes, 26 (miscellaneous) with 106 genes, and 30 (signaling) with 96 genes (Supplementary Table 2). In addition, there were about 12 bins with at least 20 or more significant genes. To perform a close up of our data and gain valuable information about the pathways and processes positively/negatively affected in our lines, we selected the metabolism overview option in MapMan to visualize metabolic perturbations (Figure 3A; Supplementary Table 3). On the one hand, the analysis revealed a general upregulation of genes involved in the processes related to secondary metabolism (e.g., waxes, terpenes, flavonoids, phenylpropanoids, and phenolics), cell wall, lipids, nucleotides, tetrapyrroles, and amino acids. On the other hand, downregulation was observed in processes such as electron transport in the mitochondria, light reactions of photosynthesis, ascorbate, and glutathione metabolism (Figure 3A). Moreover, the MapMan analysis identified 469 genes involved in biotic and abiotic stress responses (Figure 3B; Supplementary Table 4). Genes belonging to processes such as proteolysis, cell wall, abiotic stress, chaperones (HSP), secondary metabolites, heat shock proteins, and transcription factors were mainly upregulated, while genes belonging to processes such as hormone signaling and the redox state were mainly downregulated (Figure 3B). In addition, genes with regulatory functions were identified in processes such as transcription, protein modification and degradation, hormone network (IAA, ABA, BA, ethylene, cytokinin, and GA), and redox (Supplementary Figure 2A; Supplementary Table 5). Interestingly, most of the genes involved in biotic and abiotic stress (e.g., heat, cold, and drought/salt) responses were upregulated (Supplementary Figure 2B; Supplementary Table 6). Unexpectedly, 139 transcription factors, including auxin response factors (ARFs), bHLHs (basic helix-loop-helix), bZIPs (basic leucine zipper), ethylene response factors (ERFs), MYBs (oncogene from myeloblastosis virus), NACs [acronym derived from NAM (no apical meristem)], ATAF1/2, and CUC2 (cup-shaped cotyledon), which were the three initially discovered genes to contain a conserved NAC domain, and WRKYs (after the WRKY conserved amino acid sequence in the N-terminus of the protein) were significantly up- and downregulated in a constant and robust manner (Supplementary Figure 3; Supplementary Table 7).

**Figure 4:**
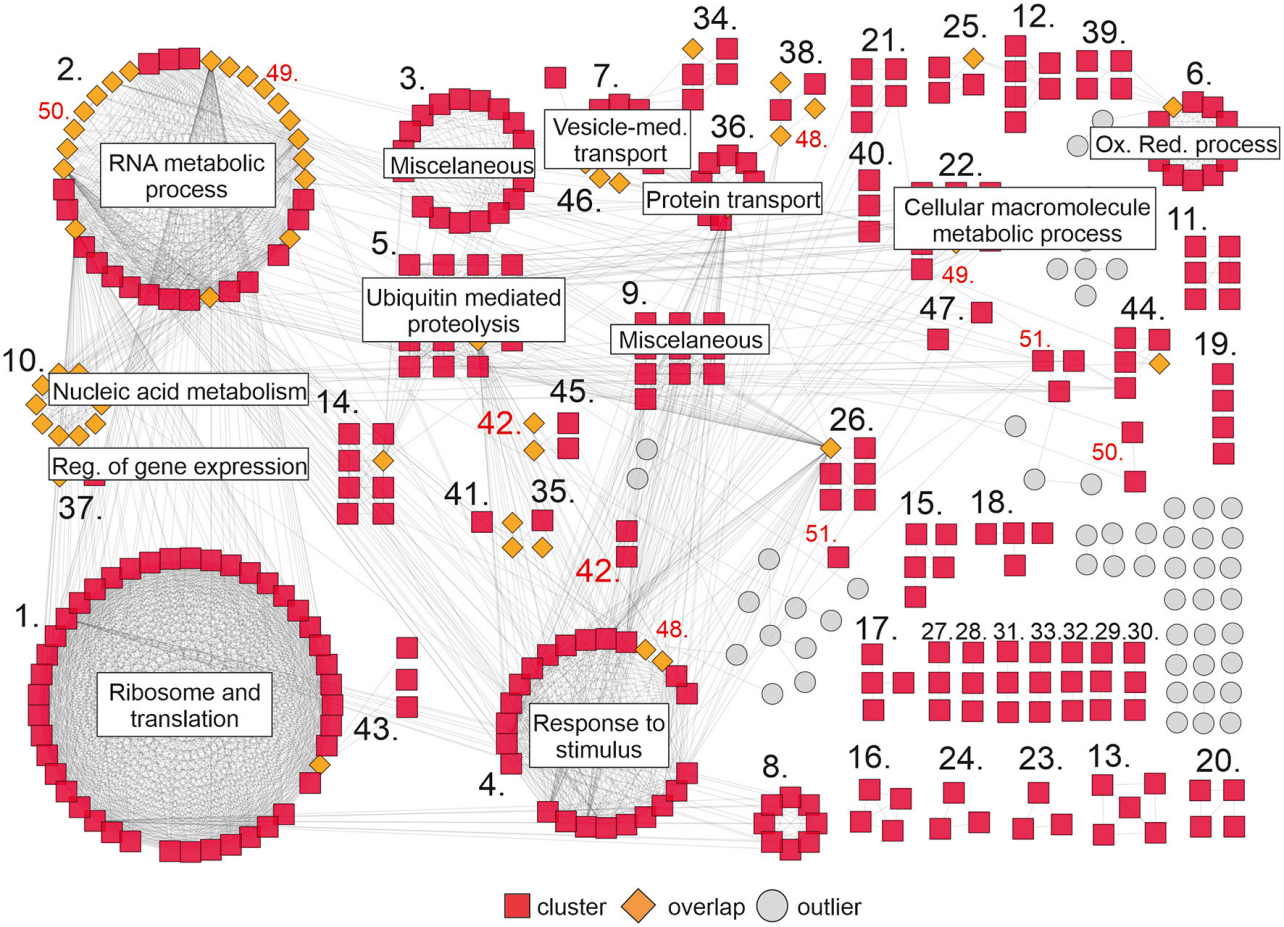
Gene network of DEGs upon *DcLCYB1* expression in tobacco transgenic lines. Gene network representation of differentially expressed genes (up- and downregulated) in *DcLCYB1* transgenic lines. The network comprises 51 clusters (including 419 interacting genes). Only clusters with 10 or more members are provided with a name in the figure (detailed cluster composition can be found in Supplementary Table 8). A cluster number in red means there are genes grouped in different locations. Gene expression data from the RNAseq experiment were used to build the network.

To complement our analysis, we built a gene network based on the interactions between the DEGs in our RNAseq analysis. Interaction data were obtained from the String database (Szklarczyk et al., 2017) and imported into Cytoscape (Shannon et al., 2003) to visualize the network. Approximately 420 genes grouped in 51 clusters interact with each other (Figure 4). The largest clusters correspond to ribosome and translation (43), RNA metabolic process (36), response to stimulus (24), ubiquitin mediated proteolysis (15), and oxidation-reduction process (12). Other smaller clusters correspond to cellular macromolecule metabolic process (10), protein transport (10), nucleic acid metabolism and the regulation of gene expression (10), vesicle-mediated transport (10), and others (Figure 4; Supplementary Table 8). Interestingly, there are some genes in the network (orange rhombus in Figure 4) that connect one or more clusters, reflecting the high degree of interaction between the components of this network.

### Proteomic Analysis of *DcLCYB1*-Expressing Lines

**Figure 5:**
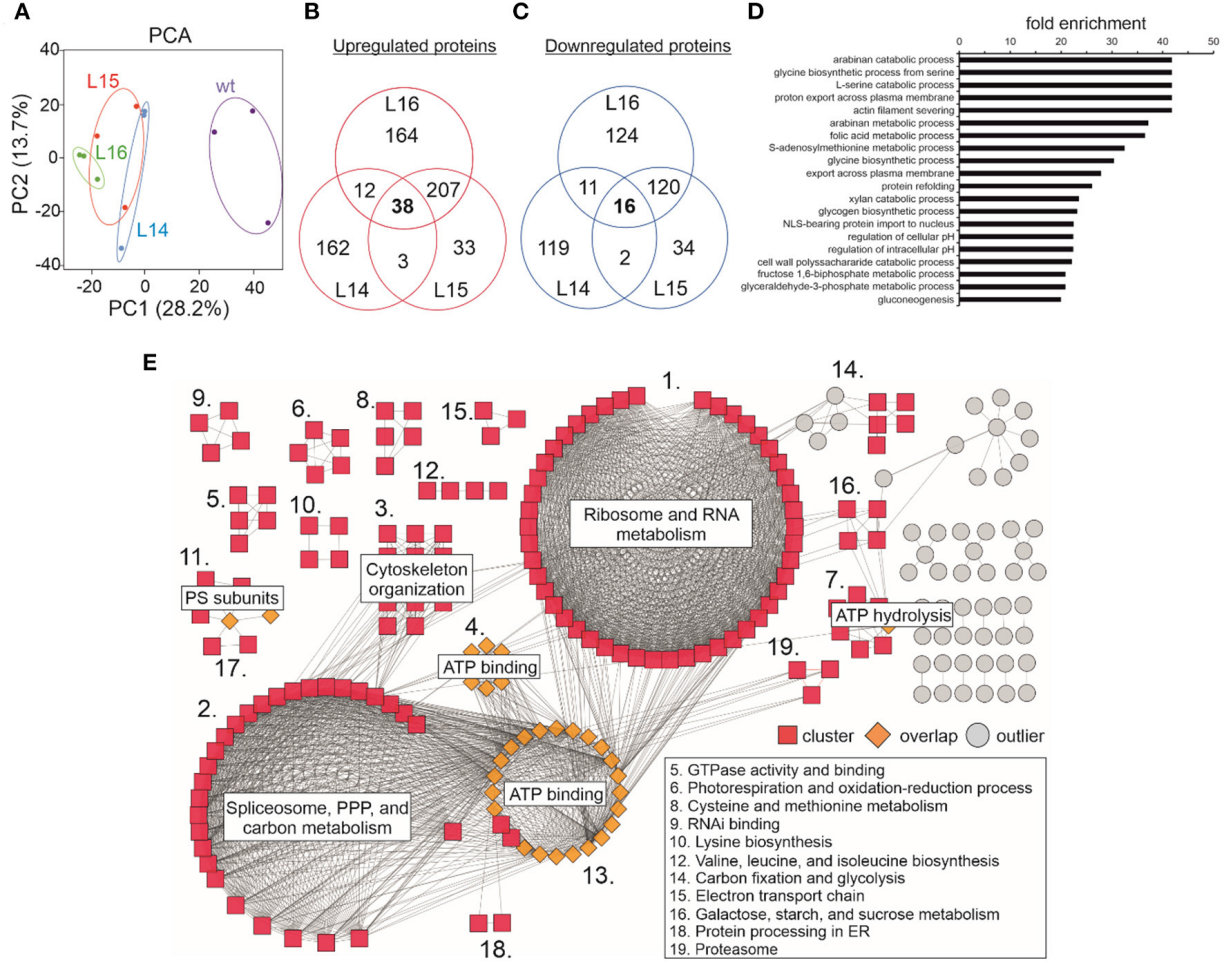
Effect of the expression of the *DcLCYB1* gene in total leaf proteome of transgenic tobacco lines. **(A)** Principal component analysis (PCA) of wild type and transgenic *DcLCYB1* lines. **(B,C)** Venn diagrams of proteins with increased and reduced abundance in the *DcLCYB1* transgenic lines (using POT data). **(D)** GO (biological process) enrichment of significantly changing (increased and reduced abundance) proteins (using the ATG codes) in at least two of the three transgenic *DcLCYB1* lines (top 20 processes are shown). **(E)** Protein network representation of proteins changing significantly (increased and reduced abundance) in the *DcLCYB1* transgenic lines. The network comprises 214 highly interacting proteins. Protein abundance was quantified by LC-MS/MS and protein intensity was log2 transformed and the linear model for microarray analysis (limma) R package (Ritchie et al., 2015) was used for comparative analysis (*p* ≤ 0.05). Three biological replicates for each genotype (wt, L14, L15, and L16) were used for shotgun proteomics (*n* = 3).

In our proteomics experiment, the results from the PCA showed that replicates within the transgenic and wild type genotypes grouped together while the differences between the wild type and transgenic groups were large (Figure 5A), suggesting a considerable change in the proteome of the transgenic lines compared with the wild type. From the ~2,900 POTs identified in our experiment (Supplementary Table 9), the chloroplast was the compartment where the highest number of proteins was identified (~31.5%; Supplementary Figure 4), followed by the cytoplasm (~15–20%), mitochondria (6.9%), and nucleus (6.9%; Supplementary Figure 4; Supplementary Table 10). This pattern was also observed for the up- (~32–35%) and downregulated (~34–35%) proteins measured for all the transgenic lines (Supplementary Table 10). Unfortunately, it was not possible to detect the *DcLCYB1* protein in our analysis. However, the endogenous *Nt*LCYB showed an increase between 10 and 30%; however, it was significant only for line L16 (Supplementary Table 9). Nevertheless, 260 proteins showed increased abundance (Figure 5B), while 149 showed decreased abundance in at least two of the three transgenic lines (Figure 5C). In addition, (GO) enrichment analysis revealed processes such as amino acid metabolic processes (e.g., glycine and serine), fructose 1,6-biphosphate and glyceraldehyde 3-phosphate metabolic processes, and gluconeogenesis (among others) with at least 20-fold enrichment (Figure 5D). In order to integrate all this information, we used the proteins overlapping in at least two lines (409) and built a protein–protein interaction network by combining the String database (Supplementary Table 11) and Cytoscape software for visualization (Shannon et al., 2003; Szklarczyk et al., 2017). Around 214 proteins showed a high degree of interaction (see the Material and Methods section; Figure 5E; Supplementary Table 12). The network revealed three main larger clusters containing the proteins involved mostly in ribosome and RNA metabolism, spliceosome, pentose phosphate pathway (PPP) and carbon metabolism, and ATP binding, respectively. Interestingly, almost all the proteins comprising the ATP binding cluster interact with proteins of the other two larger clusters, suggesting the ATP binding function as a nexus between these other two larger clusters (Figure 5E). Other smaller clusters contain the proteins involved in cytoskeleton organization, ATP hydrolysis, photosystems subunits, photorespiration and oxidation-reduction process, amino acid metabolism, RNA binding, carbon fixation, and glycolysis, among others (Figure 5E; Supplementary Table 12)

### Metabolomics Analyses Reveal Changes in Secondary Metabolites and Lipids

**Figure 6:**
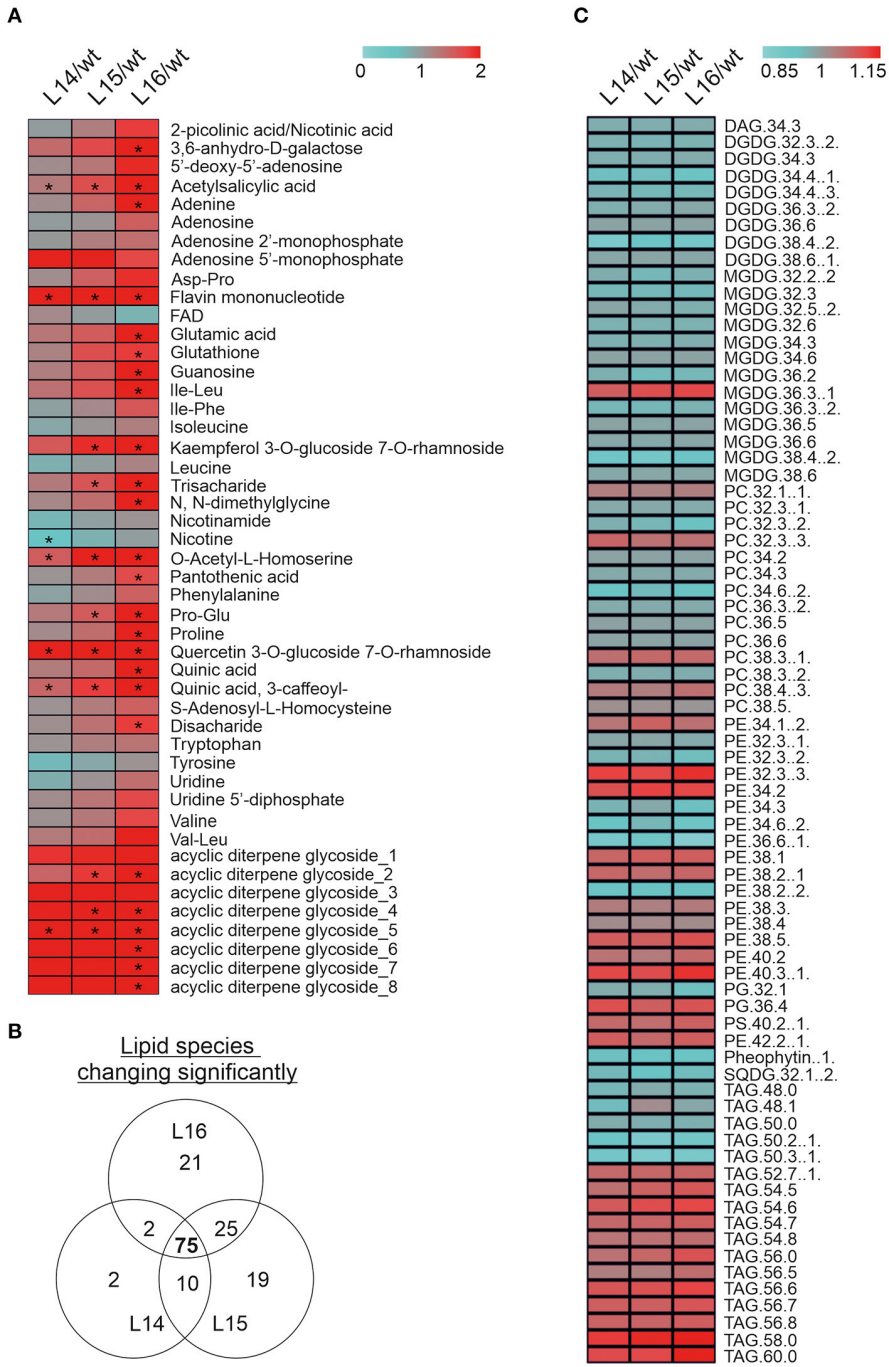
Metabolomic alterations in *DcLCYB1*-expressing tobacco lines. **(A)** The heat map represents the ratio of transgenic lines and wild type (fold change) of 46 secondary metabolites measured in the experiment. Asterisks indicate significant changes (nonpaired two-tailed student *t*-test, *p* ≤ 0.05; *n* = 6 biological replicates). **(B)** Venn diagram showing lipid number significantly changed in the transgenic *DcLCYB1* lines. **(C)** Heatmap representing the 75 of lipids changing significantly in all the transgenic lines. Significant differences were determined using nonpaired two-tailed student *t*-test (*p* ≤ 0.05; *n* = 6 biological replicates).

Because of significant changes in many of the genes and proteins involved in secondary metabolism in the transcriptome and proteome of the transgenic lines, we decided to determine changes in the secondary metabolites through a LC-MS analysis. We identified significant increases in acetylsalicylic acid, flavin mononucleotide, O-acetyl-L-homoserine, quercetin 3-O-glucoside 7-O rhamnoside, quinic acid (3-caffeoyl), and one acyclic diterpene glycoside in all transgenic lines (Figure 6A). In addition, other metabolites were increased (e.g., adenine, proline, Pro-Glu) or decreased (e.g., nicotine) in one or two transgenic lines (Figure 6A). Finally, we analyzed lipid composition in the wild type and *DcLCYB1* transgenic lines because β-carotene and polar carotenoids (xanthophylls) rigidify the fluid phase of the membranes and limit oxygen penetration to the hydrophobic membrane core, which is susceptible to oxidative degradation (Subczynski et al., 1991; Berglund et al., 1999). Thus, increases in β-carotene and xanthophylls in our transgenic lines might influence lipid composition. Interestingly, 75 lipid species were significantly changed in all three lines (Figure 6B). Small but significant decreases (~2–5%) in the lipid composition of monogalactosyldiacylglycerol (MGDG) and digalactosyldiacylglycerol (DGDG) were detected in the transgenic lines compared with the wild type (Figure 6C; Supplementary Tables 14, 15). By contrast, a general increase in phosphatidylethanolamine (PE), triacylglycerides (TAG), and phosphatidylserine (PS) was found in the transgenic lines compared with the wild type (Figure 6C; Supplementary Tables 14, 15).

### Plant Stress Tolerance Is Enhanced in *DcLCYB1*-Expressing Lines

Considering the great number of upregulated genes related to abiotic stress (Figure 3B), we decided to challenge our transgenic lines to different abiotic stresses, such as high light, salt, and oxidant agents (e.g., catechin and H_2_O_2_). We decided to asses these stresses due to the fact that enhanced xanthophyll content is reflected in enhanced photoprotection, while higher β-carotene and ABA content favor antioxidant capacity and salt tolerance. Interestingly, our transgenic lines possess the aforementioned features and therefore it is possible that they show enhance tolerance to theses abiotic stresses. Due to the increased hormone content (GAs and ABA) (Moreno et al., 2020) and altered gene expression related to hormone signaling (Figure 3B) in these lines, we first characterized the early growth and development of these lines. As expected, radicle emergence and germination were delayed in the transgenic lines compared with the wild type (Supplementary Figures 5A,B), reflecting the increased ABA content in these lines. In addition, leaf area and internode length were increased in the transgenic lines compared with the wild type in later developmental stages in plants growing in the greenhouse (Supplementary Figures 5C,D), which is well in line with the increased GA content in these lines.

**Figure 7:**
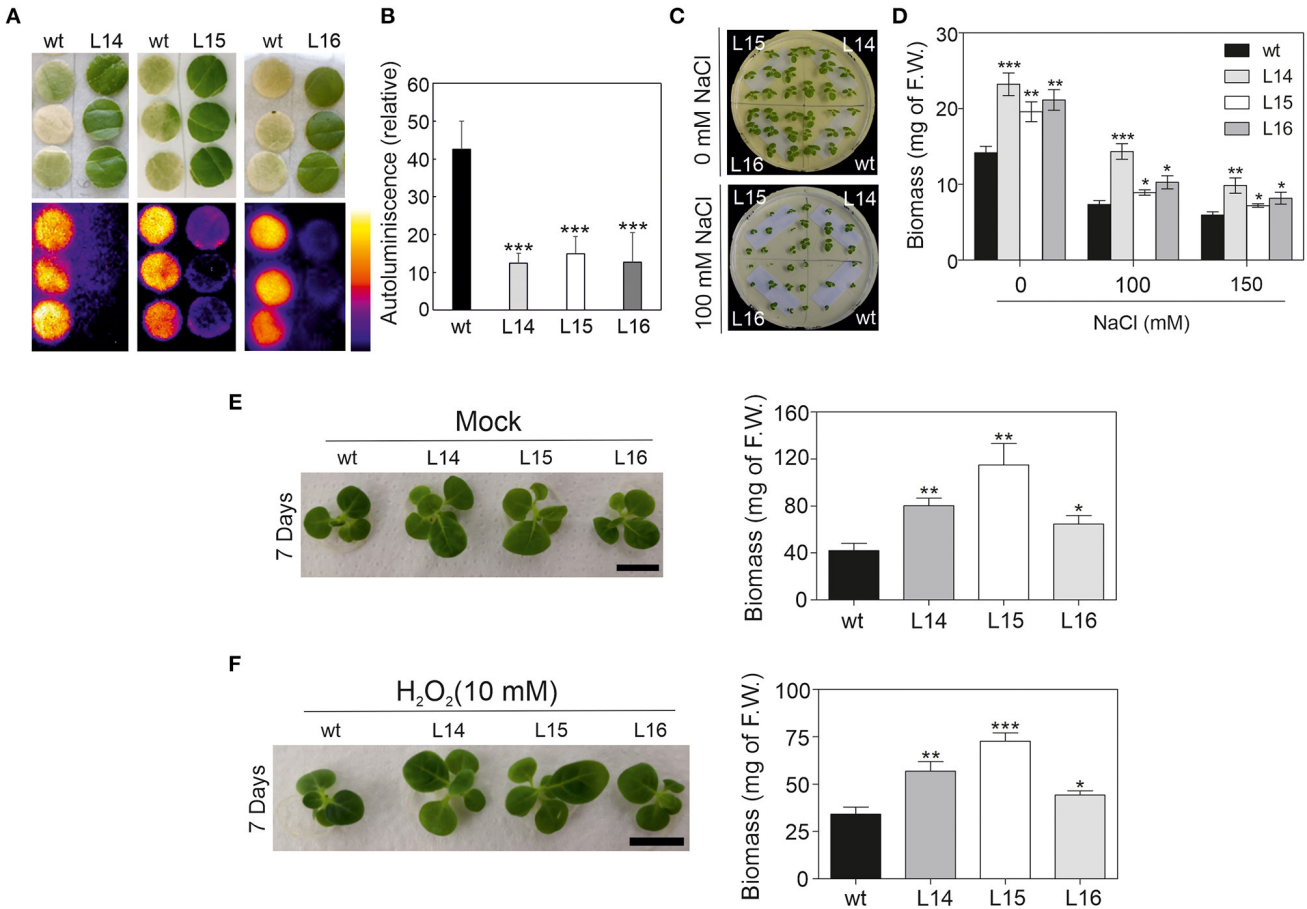
High light, salt, and oxidative stress response in *DcLCYB1* tobacco lines. **(A)** Lipid peroxidation analysis by imaging of the wild type and lines L14, L15, and L16 (*n* = 3). **(B)** Quantification of the relative autoluminiscence of the wild type and transgenic *DcLCYB1* lines after stress from a high amount of light. **(C)** NaCl control (upper panel) and treatment (100 mM; bottom panel) of wild type and transgenic lines. **(D)** Biomass quantification (F.W.) of wild type and transgenic lines at 0, 100, and 150 mM of NaCl treatment. **(E,F)** Oxidant agents (mock and H_2_O_2_) treatments of wild type and transgenic lines. Two-week-old seedlings grown in liquid MS media were transferred to liquid MS supplemented with water (mock) and H_2_O_2_ (10 mM) and photographed after seven days of treatment. Plant biomass (F.W.) of wild type and transgenic lines was recorded for mock- (E) and H_2_O_2_-treated (F) plants after seven days. Unpaired student's *t*-test was performed to compare transgenic lines with the wild type. *p < 0.05, **p < 0.005, ***p < 0.0005.

Then, we challenged our transgenic lines and exposed them to different abiotic stresses. First, tobacco leaf discz were exposed to high light intensity (see the Material and Methods section), and the autoluminescence was measured. Quantification of the autoluminescence in the leaf discz reflects the accumulation of lipid peroxidation in the leaf. The tobacco leaf discz from the transgenic lines remained green, while the wild type bleach (Figure 7A) suggesting increased tolerance to high light intensity in the transgenic lines. In addition, lipid peroxide accumulation in the transgenic lines was approximately four times lower than in the wild type (Figure 7B). Second, we exposed tobacco seedlings to different salt concentrations (100 and 150 mM) and observed their phenotype. Interestingly, all transgenic lines showed higher biomass in both salt concentrations, and L14 was the line with the highest salt tolerance (Figures 7C,D). Third, we exposed tobacco seedlings to catechin and H_2_O_2_ (Scarpeci et al., 2008; Kaushik et al., 2010), two well-characterized oxidant agents. The transgenic lines showed higher plant biomass under control (mock), catechin, and H_2_O_2_ treatments compared with the wild type (Figures 7E,F; Supplementary Figure 5E). In addition, the leaf area of cotyledons and the first four leaves were quantified in the wild type and transgenic lines under control and stress conditions. In general, the transgenic lines showed a bigger leaf area than the wild type under control conditions and under oxidative stress (Supplementary Figure 5F). Taken together, these results showed enhanced stress tolerance in the transgenic lines, which is reflected in a higher biomass under different stress conditions.

## Discussion

Carotenoids are important isoprenoid molecules involved in the essential functions for plant growth and development (e.g., photosynthesis, photoprotection, ROS-scavenging). Moreover, carotenoids (e.g., β-carotene) are the precursors of plant hormones (e.g., ABA and SLs). Thus, manipulation of carotenoid metabolism can influence hormonal content (e.g., GA, ABA) and trigger growth and developmental responses in addition to the expected changes in pigment accumulation (Moreno et al., 2020). In the current study, we showed the impact of *DcLCYB1* gene expression at the transcriptome, proteome, and metabolic levels of our previously published *DcLCYB1* tobacco lines (Moreno et al., 2020), attempting to explain how these changes support higher biomass and stress tolerance. *DcLCYB1* tobacco lines showed higher β-carotene, lutein/zeaxanthin, and violaxanthin content, which resulted in higher ABA but also indirectly in higher GA content. Increased hormone content altered plant architecture (Figures 1A,B), resulting in enhanced photosynthetic efficiency and plant yield in these lines (Moreno et al., 2020). Enhanced carotenoid and hormone content, photosynthetic efficiency, and plant yield can be very desirable traits in crops, making the genetic manipulation of the carotenoid pathway—particularly of the *LCYB* gene—a promising target for crop improvement. However, additional information at the molecular level (transcriptome, proteome, and the overall metabolism) is needed to complete the analysis of these lines before exporting this bioengineering to crops.

**Figure 8:**
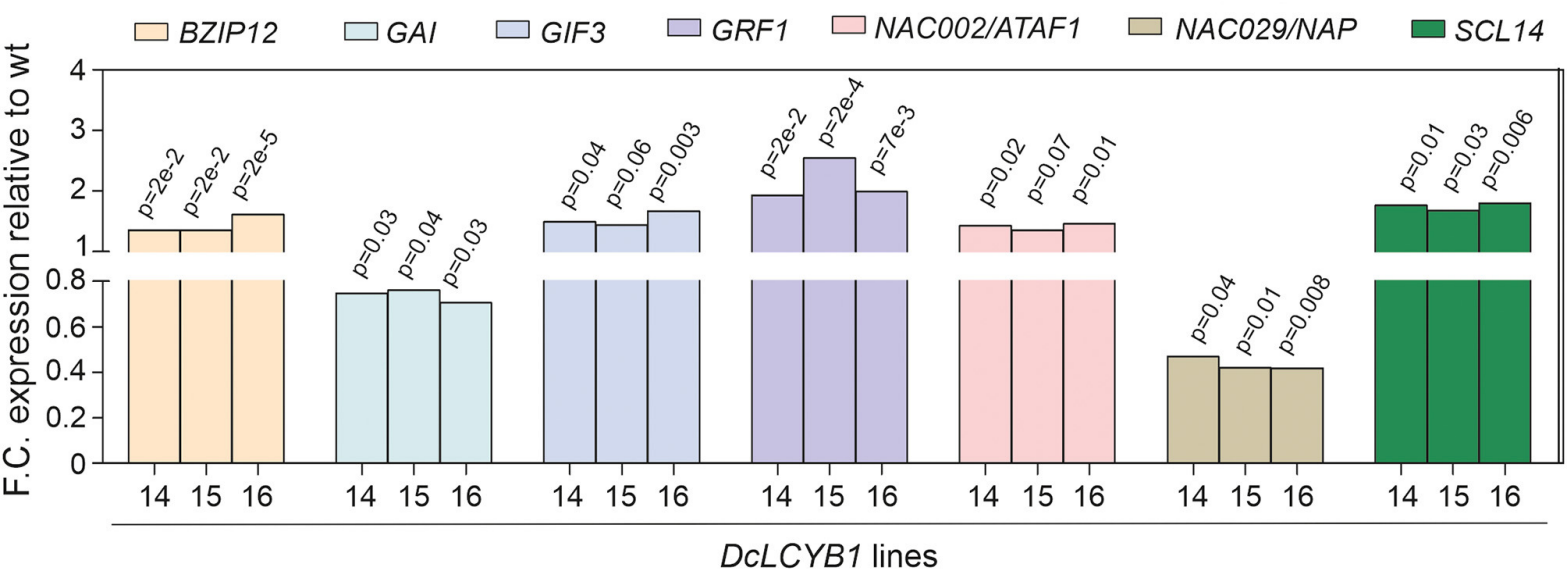
Fold change expression of transcription factors involved in growth, development, and stress response. Transcription factors involved in light and salt responses and in plant growth and development. Gene expression was measured by RNAseq (padjust < 0.05). e: represents 10.

At the transcriptome level, *DcLCYB1* expression caused genetic changes that extended beyond the chloroplast (e.g., cytosol, nucleus; Supplementary Figure 1), which is the organelle where the LCYB protein is located and where it plays a key role in carotenoid synthesis. Moreover, the expression of ~2,000 genes changed significantly in the transgenic lines, raising the question of how a single gene transformation with a gene encoding an enzyme of the carotenoid pathway can trigger this response. Interestingly, the cytosol and nucleus were the other compartments with the highest number of genes that changed significantly (Supplementary Figure 1). The cytosol and nucleus are compartments in which transcription factors are localized and can induce changes in gene expression that favor stress tolerance, growth, and development (Aida et al., 1997; Kim et al., 2003; Tran et al., 2004; Wu et al., 2009; Beltramino et al., 2018; D'Alessandro et al., 2018). This suggests a plastid-to-nucleus communication that could support the ~2,000 genes changing significantly in the transgenic lines. In fact, consistent changes in the expression of transcription factors, which could influence the expression of a large number of genes, were detected in our transgenic lines (Supplementary Table 7). For instance, the expression of the *bZIP12* transcription factor increased ~40% in the transgenic lines (Figure 8). This transcription factor was reported to enhance salt tolerance in rice (Hossain et al., 2010) and is in line with the enhanced salt tolerance shown in our transgenic lines. In addition, a NAC [ATAF1 (activating factor 1)/NAC002] transcription factor was shown to mediate the responses to abiotic stress in Arabidopsis (Wu et al., 2009). Moreover, ATAF1 regulates and promotes salt tolerance in rice (Liu et al., 2016). In rice, the *ATAF1* overexpressors, the *DEHYDRIN* and *LEA* (e.g., *OsLEA3*) genes, are induced. These genes contribute to the salt tolerance mechanism in rice and Arabidopsis (Chourey et al., 2003). In our transgenic lines, upregulation of *DEHYDRIN* (~200% increase) and *LEA* (230% increase) as a response to increased *ATAF1* expression (~40%; Figure 8) might have contributed to the enhanced salt tolerance in our plants. In addition, *ATAF1* is a positive regulator of ABA synthesis (Jensen et al., 2013). Indeed, enhanced *ATAF1* expression in our transgenic lines is in line with the observed increase in ABA content (Moreno et al., 2020) and enhanced salt tolerance. Another NAC family member (NAP/NAC029) was reported to be involved in salt tolerance, GA-mediated chlorophyll degradation, and leaf senescence (Seok et al., 2017; Lei et al., 2020). Arabidopsis *nap* mutants showed enhanced salt tolerance in plants grown using synthetic media and using soil (Seok et al., 2017). Reduced *NAP* expression (~55%; Figure 8) in our transgenic lines might contribute to the enhanced salt tolerance observed in plants grown on synthetic media supplemented with NaCl (Figures 7C,D). Moreover, *NAP* expression promotes leaf senescence and GA-mediated chlorophyll degradation (Lei et al., 2020). Thus, the reduced NAP expression in our transgenic lines might contribute to the observed delayed senescence phenotype and enhanced chlorophyll content found in our tobaccos (Moreno et al., 2020).

The SCARECROW-LIKE protein (SCL14) is a member of the Arabidopsis GRAS family of transcription factors. SCL14 competes with GRX480/ROXY19 for binding the TGAII transcription factors and mediating the activation or inhibition, respectively, of the detoxification response (Ndamukong et al., 2007; Fode et al., 2008; Koster et al., 2012; Huang et al., 2016). Recently, it was shown that SCL14-dependent detoxification is necessary for the resilience of Arabidopsis plants exposed to photooxidative stress (D'Alessandro et al., 2018). In addition, Arabidopsis *scl14* mutants with reduced and enhanced *SCL14* expression showed reduced and enhanced tolerance, respectively, to high light (D'Alessandro et al., 2018). It was shown that β-cyclocitral (β-cc) induces the expression of SCL14, which interacts with TGAII and activates the xenobiotic detoxification response through the NAC002/ATAF1 transcription factor (D'Alessandro et al., 2018; D'Alessandro and Havaux, 2019). Interestingly, our transgenic lines showed enhanced tolerance to high light (Figures 7A,B). Moreover, increased expression of the SCL14 (~80%) and NAC002/ATAF1 (~40%) transcription factors suggest the activation of the xenobiotic response, resulting in higher tolerance to high light. However, β-cc content did not change in our transgenic lines (Moreno et al., 2020), suggesting the possibility of a β-cc-independent activation of the xenobiotic response. Other members of the GRAS family also showed increased expression in our dataset (SCL9 and SCR), which might be contributing to the high tolerance to high light in our transgenic lines.

Another interesting example is the gibberellic acid insensitive (GAI), which represses GA responses and restrains the normal cell proliferation and expansion that drive plant growth. In our transgenic lines, growth is enhanced because of an increase in GA levels (Moreno et al., 2020). This is correlated with reduced *GAI* gene expression identified by RNAseq in our transgenic lines (Figure 8). Reduction of *GAI* allowed for the accumulation of GA, resulting in enhanced plant growth. Furthermore, *GRF (growth regulating factor)-interacting factor3* (*GIF3*) is a member of a small family of transcription coactivators that forms functional complexes with GRFs (Kim et al., 2003; Lee et al., 2009; Lee and Kim, 2014). *GIF3* is required for the cell proliferation activities of lateral organs, including leaves and cotyledons (Kim et al., 2003; Lee et al., 2009). The Arabidopsis *gif1* mutant showed reduced leaf and cotyledon size (Kim and Kende, 2004). Increased *GIF3* expression by ~50% in our transgenic lines supports the observed increased size in cotyledons and leaves under control and stress conditions (Supplementary Figures 5C,F). In the same line of evidence, a GRF (*GRF1*) involved in leaf and cotyledon growth (Kim et al., 2003) was detected in our RNAseq analysis. Arabidopsis mutants with reduced and increased GRF expression showed reduced and increased leaf and cotyledon size, respectively (Kim et al., 2003; Kim and Kende, 2004; Beltramino et al., 2018). Thus, increased *GRF1* expression (up to 150%) in our transgenic lines (Figure 8) might contribute to the enhanced growth observed in leaves and cotyledons under normal and stress conditions in our tobacco lines (Supplementary Figures 5C,F). In a recent study, an RNAseq analysis showed a broad range of gene targets for GRF1 and GRF3, including the genes involved in plant growth and development, phytohormone biosynthesis and signaling, and the cell cycle (Piya et al., 2020). Moreover, clock core genes and genes with stress- and defense-related functions are the most predominant among the GRF1 and GRF3-bound targets. Additionally, it was shown that GRF1 and GRF3 target molecular nodes of growth-defense antagonism and modulate the levels of defense- and development-related hormones (e.g., ABA) (Piya et al., 2020). This is in line with our RNAseq analysis (Figure 3B; Supplementary Figures 2A,B), where the expression of more than 100 genes involved in hormone biosynthesis and signaling, cell division, cell cycle, and development was significantly changed in our transgenic lines.

Interestingly, many of the affected processes at the transcript level (RNAseq) were also identified at the proteome level. For instance, the biggest nodes in the protein–protein interaction network correspond to ribosome and RNA metabolism, which comprises the proteins involved in transcription and translation (e.g., ribosomal subunits). The reshaping in those processes might be indicators of higher translation to support the increased/decreased abundance of around 600 proteins in the transgenic lines. Proteins belonging to processes such as photorespiration and redox, amino acid metabolism and biosynthesis, RNA binding, carbon fixation and glycolysis, and electron transport chain reflect a direct impact on the increased/reduced gene expression measured in our RNAseq experiment (Figures 4, 5; Supplementary Tables 2–6, 10–12).

**Figure 9:**
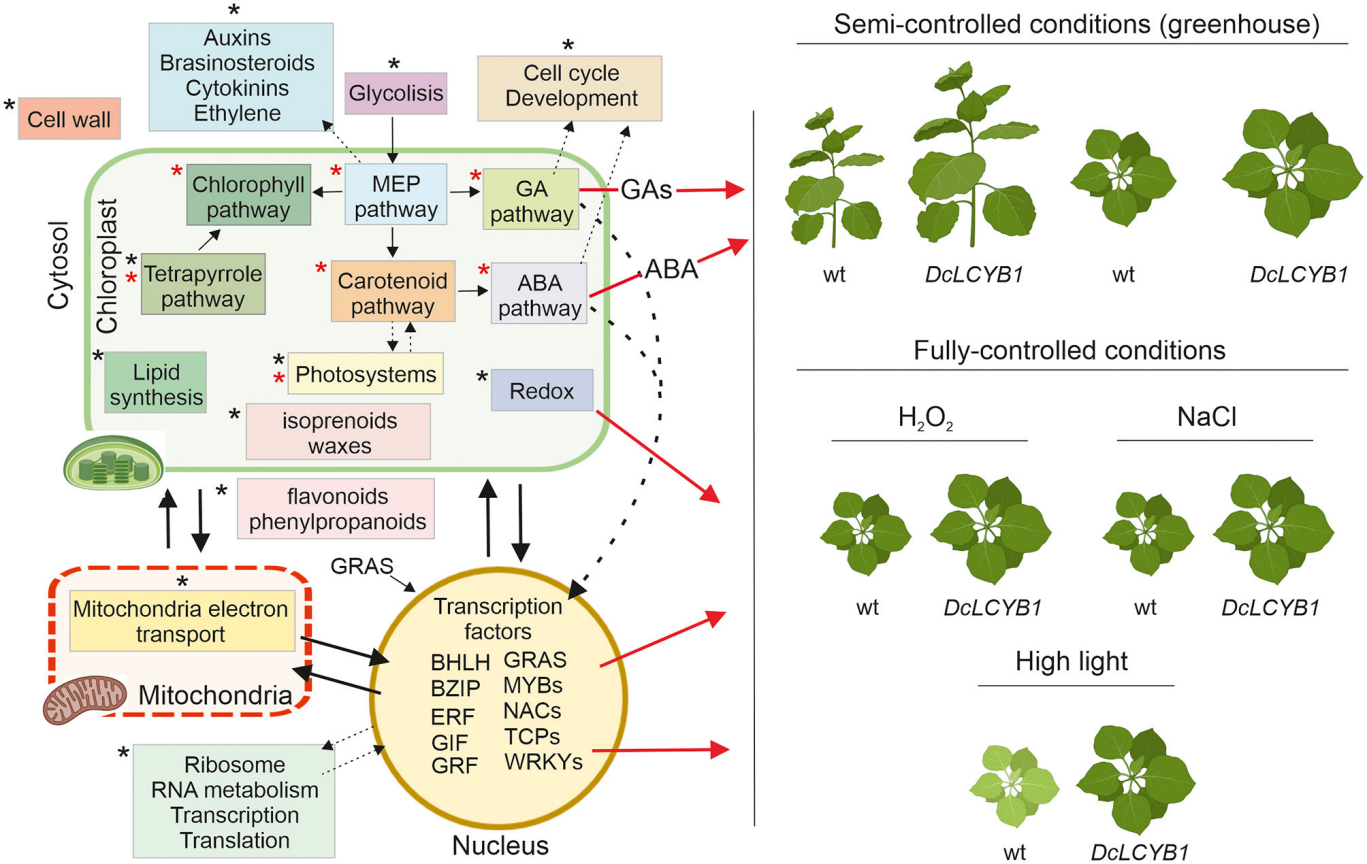
Schematic model representing pathways and processes with changes at transcript, protein, and metabolic level and allowing greater plant biomass and stress resilience in tobacco *DcLCYB1* lines. Expression of *DcLCYB1* results in a reshaping of the tobacco transcriptome, proteome and metabolome affecting processes outside the chloroplast (e.g., mitochondria, nucleus, and cytosol). Previously reported processes that were affected upon expression of *DcLCYB1* (Moreno et al., 2020) are indicated with a red asterisk, while newly identified processes (altered upon *DcLCYB1* expression) with our OMICs strategy are indicated with a black asterisk. Although increased hormone content (e.g., ABA) might have enhanced stress tolerance, here we integrate the genes and proteins that can influence the expression of other genes (e.g., transcription factors) and can trigger signaling cascades involved in stress tolerance, growth, and development. A schematic representation using *Nicotiana benthamiana* plants (available in Biorender) were used to represent the stress response in the *DcLCYB1* lines. The model was build using Biorender (Biorender.com).

Summarizing and integrating our results, we provide a model that explains the higher yield phenotype and high light, salt, and oxidative stress tolerance observed in our transgenic tobacco lines beyond the previously reported isoprenoid pathways in chloroplast (Figure 9). The carotenoid and carotenoid-related pathways influencing phytohormone content or photosynthesis and, thus, impacting plant growth (red asterisks in Figure 9) were thought to be the main reason for the higher yield phenotype (Moreno et al., 2020). However, by combining transcriptome, proteome, and metabolome data, we have provided new evidence suggesting other processes and pathways (black asterisks in Figure 9), for instance, in the cytosol and nucleus, are involved in this phenomenon. One example is the increased gene expression and protein abundance of glycolytic enzymes, which might be related to the increased GA, carotenoid, and chlorophyll content observed in our tobacco lines. Glycolysis provides the precursor used for isoprenoid synthesis in the MEP pathway, and MEP provides the common precursor (GGPP) for GA, carotenoids, and chlorophyll synthesis. More MEP precursors could lead to higher GGPP content, thus supporting increases in MEP-derived isoprenoid pathways. On the one hand, a great number of genes and proteins involved in cell cycle and development (Supplementary Tables 6, 9) might contribute to the observed growth rate and accelerated development in our tobacco lines. In addition, the transcription factors involved in plant growth and development (transcription factors GIF3 and GRF1, and GAI growth-related factor) might also contribute to the high-yield phenotype observed under control conditions. Furthermore, the DEGs and proteins involved in auxin, brassinosteroid, cytokinin, and ethylene metabolism might indicate their participation in growth and developmental phenotypes observed in our lines. On the other hand, besides the higher ABA content, the significant changes in transcription factors (localized in the nucleus and cytoplasm) involved in salt (bZIP12, ATAF1, NAP) and tolerance to high light (GRAS SCL14, ATAF1) together with a reshaping at the transcriptome and proteome level of the redox process contribute to the observed higher tolerance to high light, salt, and oxidative stress in our transgenic tobacco lines (Figure 9). Interestingly, previous studies have shown that altered hormone content (e.g., ABA) induces the expression of transcription factors and vice versa (Devkar et al., 2020; Piya et al., 2020; Yu et al., 2020), thus activating the signaling cascades that impact plant growth, development, and stress response.

In conclusion, what seemed to be a simple expression of a carotenogenic gene that encodes for an enzyme converting the lycopene into β-carotene resulted in a greater reshaping at transcriptome, proteome, and metabolome levels in different cell compartments to support plant growth, development, and stress tolerance in tobacco. The progress and knowledge generated here will allow us to take this bioengineering to the next level, applying it to crops to generate a new generation of super crops with enhanced photosynthetic efficiency, yield, stress tolerance, and nutritional content.

## Data Availability Statement

The datasets presented in this study can be found in online repositories. The names of the repository/repositories and accession number(s) can be found in the article/Supplementary Material.

## Author Contributions

JM and AS conceived the project and wrote the manuscript with input from the coauthors. JM performed the cDNA synthesis, metabolite extraction, and MapMan analysis and analyzed RNAseq data. JM and MK performed the oxidative stress experiments. PS performed salt stress experiments. ES run samples for proteomics and the data deposition. SM-J and JM performed statistical analysis and analyzed and interpreted the data. AS performed the secondary metabolite data analysis. MH performed the photooxidative stress and lipid peroxidation experiments. AF performed the mapping of the NiTAB identifiers into Arabidopsis AGI codes and data deposition. UL performed lipid data analysis. All authors contributed to the article and approved the submitted version.

## Conflict of Interest

The authors declare that the research was conducted in the absence of any commercial or financial relationships that could be construed as a potential conflict of interest.
