# Supplementary material for: A Multi-OMICs Approach Sheds Light on the Higher Yield Phenotype and Enhanced Abiotic Stress Tolerance in Tobacco Lines Expressing the Carrot lycopene β-cyclase1 Gene
Source: Front Plant Sci. 2021 Feb 5;12:624365. doi: 10.3389/fpls.2021.624365 (PMC7893089; doi:10.3389/fpls.2021.624365)
Supplement: Supplementary file 1 [file Image_1.pdf]

Supplementary Figures

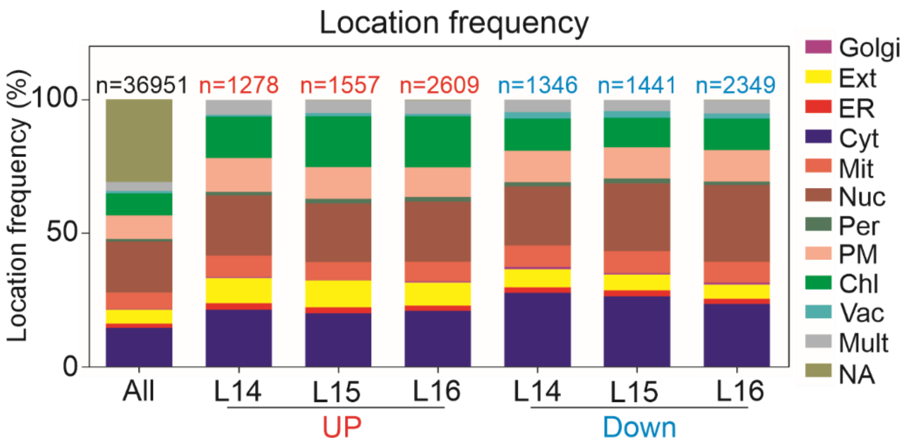

Supplementary Figure 1. In silico subcellular localization of DEGs. Location frequency analysis was performed for the total and the significantly up- and downregulated genes identified in the RNAseq experiment. Subcellular localizations of the protein encoded by each gene identified in the RNA sequencing were annotated using the SUBA3 database (http://suba3.plantenergy.uwa.edu.au/). Multiple localizations (two or more) were grouped in multiple locations (Mult). Chl, chloroplast; Cyt, cytosol; Mit, mitochondria; Nuc, nucleus; Ext, extracellular; Per, peroxisome; Vac, vacuole; ER, endoplasmic reticulum; PM, plasma membrane; N.A., not annotated in Arabidopsis.

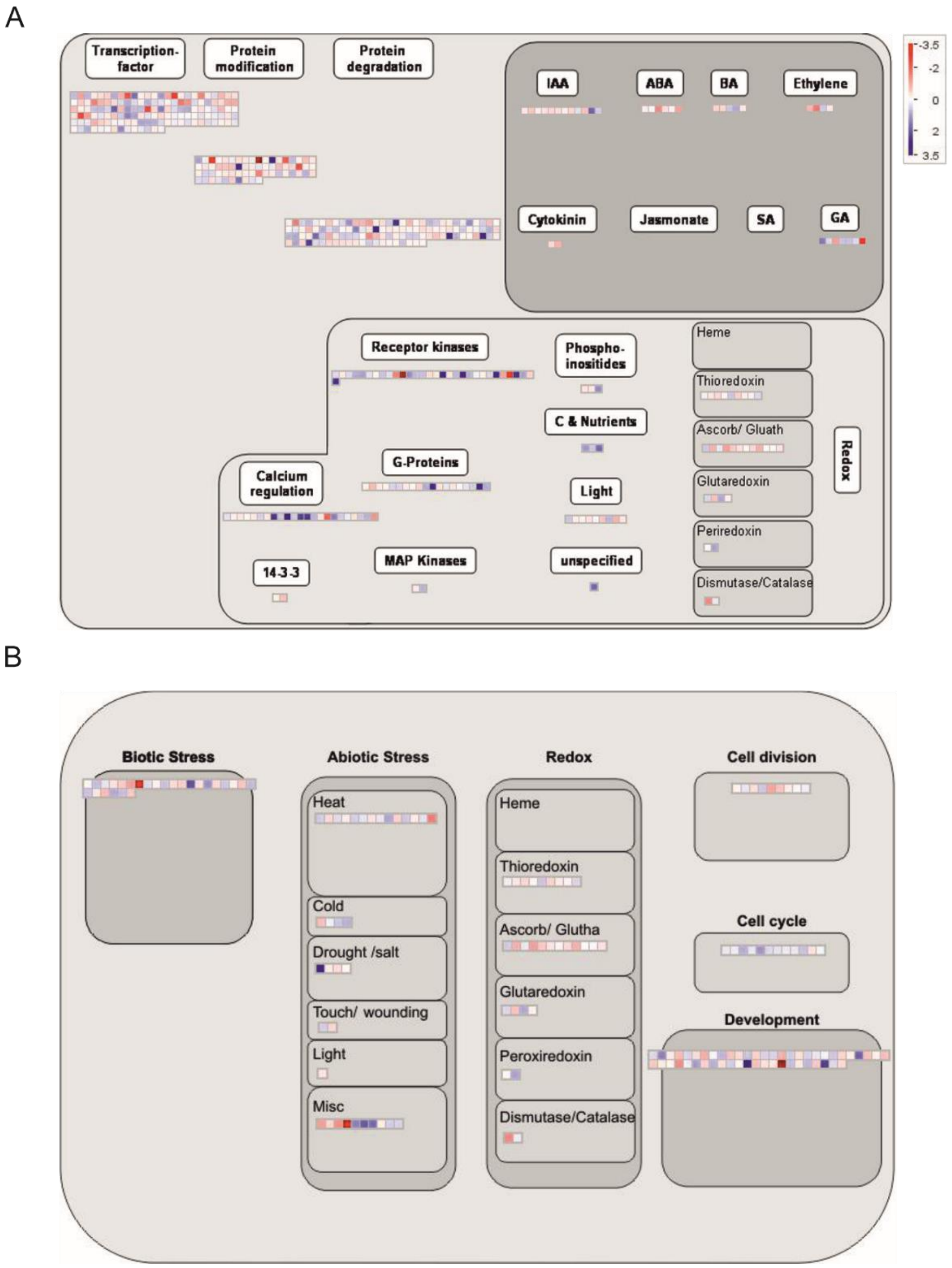

Supplementary Figure 2. MapMan representation of transcriptional perturbations in *DcLCYB1*-expressing plants. (A) Regulation overview. **(B)** Cellular response.

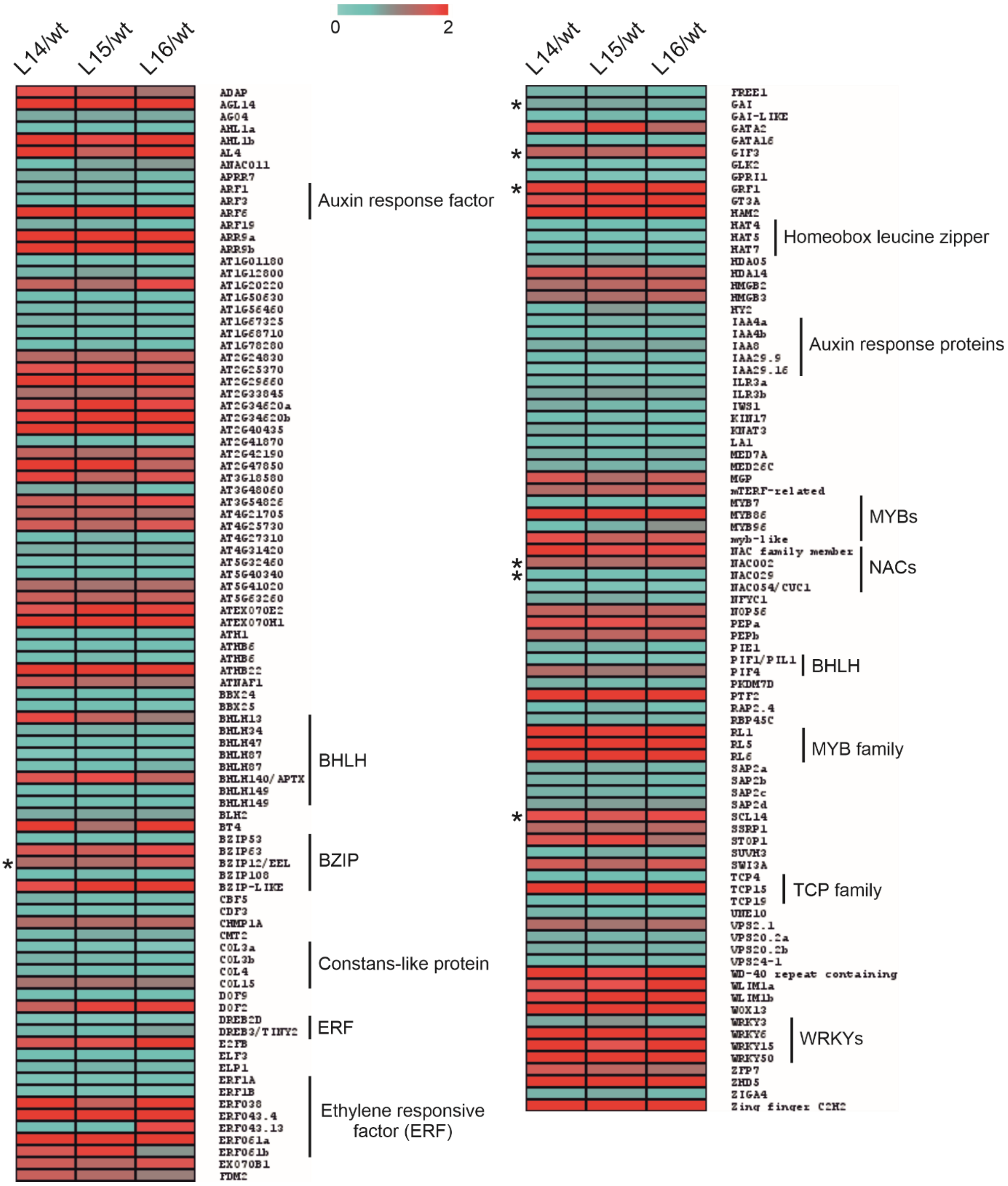

Supplementary Figure 3. Transcription factors changing significantly in the *DcLCYB1* lines. Heatmap representation of transcription factors that were significantly up- and downregulated in the transgenic *DcLCYB1* tobacco lines. The changes in gene expression were measured by RNA sequencing (padjust<0.05). Asterisks indicate the transcription factors selected to prepare **Figure 8A**.

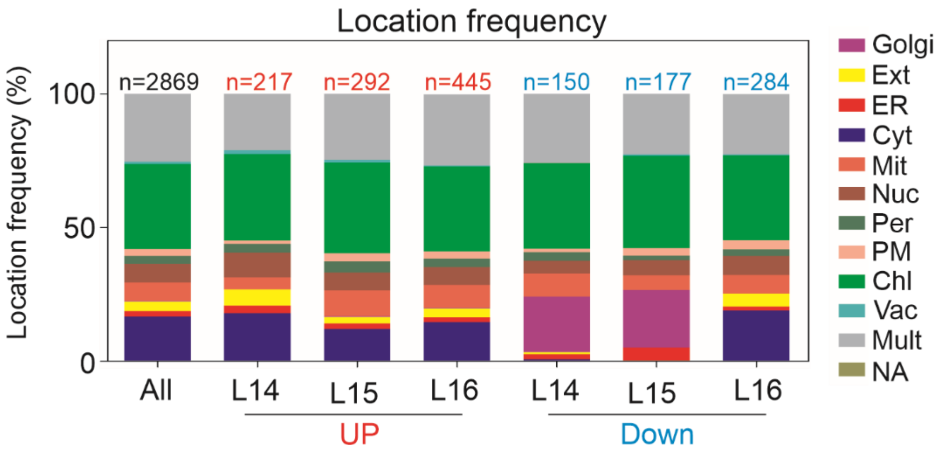

Supplementary Figure 4. *In silico* subcellular localization of proteins identified through shot-gun proteomics in the transgenic *DcLCYB1* tobacco lines. Cellular compartment distribution of identified, up- and downregulated proteins comprising the entire (detected and identified) leaf proteome of the transgenic *DcLCYB1* lines. Subcellular localizations for each protein were identified using SUBA3 database (http://suba3.plantenergy.uwa.edu.au/). Multiple localizations (two or more) were grouped in multiple locations (Mult). Homologs in *Arabidopsis* for each identified POT were retrieved and further used for localization analysis. Chl, chloroplast; Cyt, cytosol; Mit, mitochondria; Nuc, nucleus; Ext, extracellular; Per, peroxisome; Vac, vacuole; E.R., endoplasmic reticulum; PM, plasma membrane; N.A., not annotated in *Arabidopsis*.

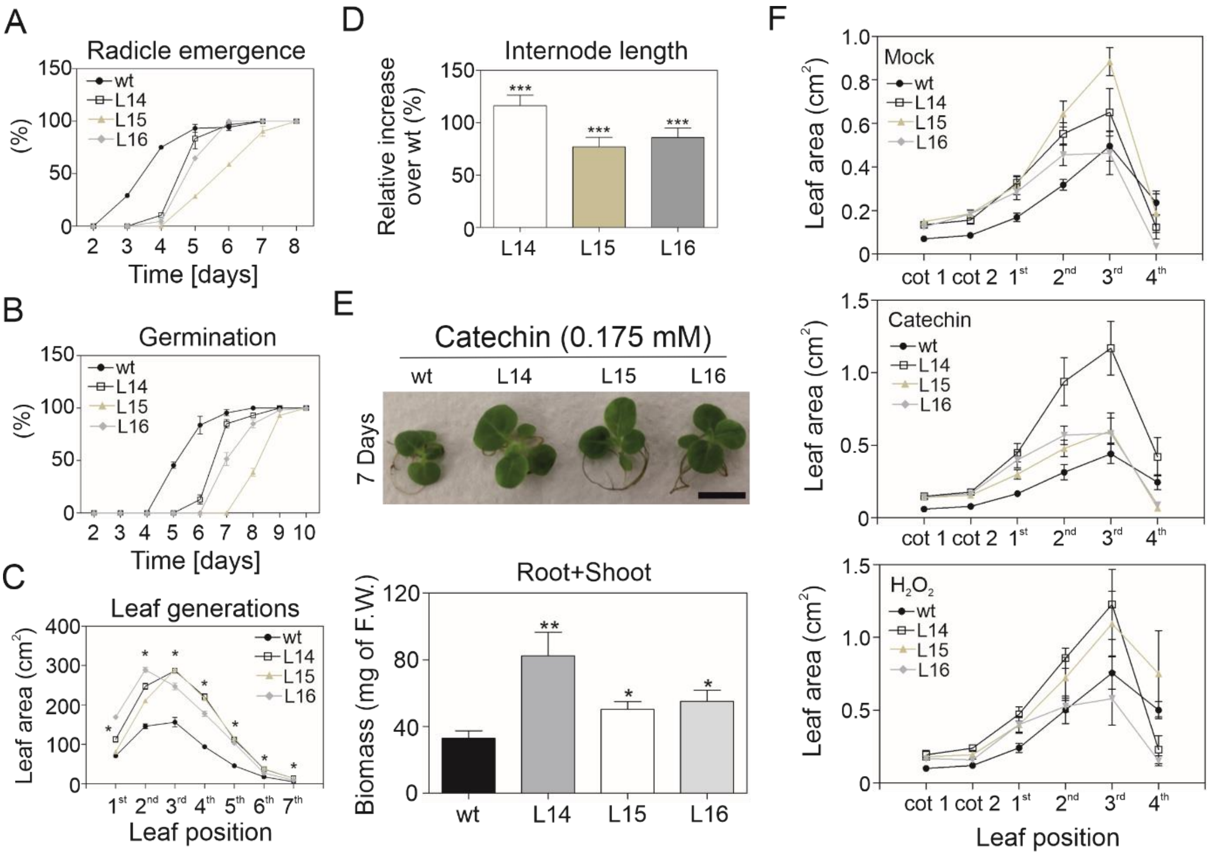

Supplementary Figure 5. Physiological parameters and oxidative stress response in *DcLCYB1* tobacco lines. (A-B) Radicle emergence and seed germination of wild type and transgenic *DcLCYB1* lines. Experiment was carried out in three different square Petri dishes with 20 seeds of each genotype per Petri dish (n = 20). **(C-D)** Leaf generations and internode length of 40-day old tobacco wild type and transgenic lines. ImageJ software was used to quantify the leaf area. Asterisks show increased leaf area from the second to the seventh leaf generation in all transgenic lines compared with the wild type (n=3). **(E)** Catechin (oxidant agent) treatment of wild type and transgenic lines. Two-week-old seedlings grown in liquid MS media were transferred to liquid MS supplemented with water (mock) or catechin (0.175 mM) and plants were photographed after seven days of treatment (upper panel). Plant biomass (F.W.) of wild type and transgenic lines after seven days of treatment (bottom panel). **(F)** Leaf area quantification of the tobacco leaf generations after exposure to mock, catechin and H_2O_2 for seven days. ImageJ was used for leaf area quantification. Unpaired student's t-test was performed to compare transgenic lines with the wild type. *: $p < 0.05$, **: $p < 0.005$, ***: $p < 0.0005$.
